# Clinical Response to Upadacitinib in Treatment-Experienced Patients With Atopic Dermatitis: A Case Series From Latin America

**DOI:** 10.7759/cureus.118166

**Published:** 2026-10-19

**Authors:** Ana B Crocker Sandoval, Yocelin N Lozano Figueroa, Ana G Perez-Romero, Alan O Muro-Davalos

**Affiliations:** 1 Dermatology, Hospital San Javier, Guadalajara, MEX; 2 Pediatric Dermatology, Hospital Civil de Guadalajara, Guadalajara, MEX; 3 Dermatology, Dermika Centro Dermatológico Láser, Guadalajara, MEX; 4 Dermatology, Antiguo Hospital Civil de Guadalajara "Fray Antonio Alcalde", Guadalajara, MEX

**Keywords:** atopic dermatitis, dupilumab, jak1 inhibitor, latin america, pruritus, real-world evidence, treatment response, upadacitinib

## Abstract

Atopic dermatitis (AD) is a chronic inflammatory skin disease that may require systemic therapy in patients with persistent disease activity or an inadequate response to previous treatments. Upadacitinib, a selective Janus kinase 1 inhibitor, has demonstrated rapid clinical efficacy in patients with moderate-to-severe AD; however, real-world data from Latin American populations remain limited. We describe the clinical responses and treatment outcomes of five adult patients with AD from Mexico who received oral upadacitinib after previous treatment with topical and/or systemic therapies. Clinical response was assessed using the Eczema Area and Severity Index (EASI), Numerical Rating Scale (NRS) for pruritus, and Dermatology Life Quality Index (DLQI) at baseline and week 2, with subsequent follow-up ranging from six months to two years. Four patients initiated upadacitinib 15 mg once daily, whereas one initially received 30 mg once daily and was subsequently reduced to 15 mg because of elevated liver enzyme levels. At week 2, all five patients experienced clinical improvement, with EASI reductions ranging from 40% to 54.5%, NRS reductions of 50-80%, and DLQI scores decreasing to 1-3. All patients maintained clinical improvement during follow-up and ultimately achieved an EASI score ≤3 and a DLQI score of 0. Notably, two patients who had previously experienced disease relapse during dupilumab treatment demonstrated marked improvement after switching to upadacitinib. One patient developed elevated liver enzyme levels approximately 1.5 times the upper limit of normal, which resolved after dose reduction; no other adverse events were reported. These cases highlight the potential role of upadacitinib in treatment-experienced patients with AD, including those with disease relapse following dupilumab treatment. Larger prospective studies are needed to further evaluate its effectiveness and safety in Latin American populations.

## Introduction

Atopic dermatitis (AD) is a chronic, multifactorial, relapsing inflammatory skin disease characterized by pruritus, epidermal barrier dysfunction, and immune dysregulation. Its prevalence is approximately 20% in children and 1-3% in adults, with a higher incidence in females [1]. AD is an immunologically heterogeneous disease driven predominantly by T helper 2 (Th2) and T helper 22 pathways, with variable contributions from T helper 1 and T helper 17 responses. Several cytokines involved in its pathogenesis, including IL-4, IL-5, IL-13, IL-22, and IL-31, signal through the Janus kinase (JAK)-signal transducer and activator of transcription pathway [2].

Patients with persistent disease, a significant impact on quality of life, or extensive disease may require systemic therapy when adequate control cannot be achieved with topical treatment. Current treatment strategies increasingly emphasize not only improvement in objective disease severity but also meaningful improvement in quality of life and sustained disease control [3].

The therapeutic landscape of moderate-to-severe AD has expanded considerably with the introduction of targeted small molecules, such as JAK inhibitors. Preclinical evidence suggests that JAK1 inhibition can reduce chronic itch through mechanisms involving Th2 cytokines, which may also directly activate sensory neurons and contribute to the perception of pruritus. These findings support JAK1 inhibition as a therapeutic strategy capable of targeting both cutaneous inflammation and chronic itch in AD [2]. Upadacitinib is a reversible, selective JAK1 inhibitor that interferes with intracellular signaling pathways involved in inflammatory responses associated with AD. It has been approved by the Food and Drug Administration for several diseases, including rheumatoid arthritis (2019), psoriatic arthritis (2021), ulcerative colitis, ankylosing spondylitis, and nonradiographic axial spondyloarthritis (2022), Crohn’s disease (2023), and giant cell arteritis (2025). Upadacitinib was approved in January 2022 for the treatment of refractory, moderate-to-severe AD in adults and children aged 12 years or older who weigh at least 40 kg [4]. Randomized clinical trials have demonstrated clinically meaningful efficacy with once-daily upadacitinib, including substantial improvements in Eczema Area and Severity Index (EASI) [5] scores with the 15-mg and 30-mg regimens [6,7]. Long-term data have shown that the safety profile of upadacitinib remains consistent with its established profile, with no new safety signals identified after up to six years and more than 9,000 patient-years of exposure [8].

We present five adult patients with AD from Mexico who received upadacitinib after previous treatment with topical and/or systemic therapies. Four patients initiated treatment with upadacitinib at 15 mg once daily, whereas one patient initially received 30 mg once daily and was subsequently reduced to 15 mg because of elevated liver enzyme levels. All patients had previously been vaccinated against herpes zoster with the recombinant zoster vaccine (Shingrix). We describe their clinical responses at week 2 and sustained disease control during follow-up ranging from six months to two years.

## Case presentation

Case 1

A 33-year-old man with a history of AD and ulcerative colitis presented with persistent cutaneous disease despite previous treatment with systemic and topical corticosteroids, sulfasalazine, and mesalamine, with only partial improvement. Dupilumab was subsequently initiated, resulting in an excellent initial cutaneous response. However, six months later, the patient experienced a relapse of AD. On physical examination, erythematous papules coalescing into several plaques were noted on the back, buttocks, and legs. At that time, the EASI score [5] was 30, the Numerical Rating Scale (NRS) score [9] was 8, and the DLQI score [10] was 26. Treatment was switched to oral upadacitinib at 30 mg once daily. At week 2, the EASI score decreased by 50%, while the NRS and DLQI scores improved by 50% and 88.5%, respectively. At six months, elevated liver enzyme levels approximately 1.5 times the upper limit of normal were detected. Therefore, the dose was reduced to 15 mg once daily, resulting in resolution of the laboratory abnormality. After two years of follow-up, EASI, DLQI, and NRS scores had decreased by 90%, 100%, and 87.5%, respectively. The clinical response was maintained throughout follow-up (Figure 1A-1C).

**Figure 1:**
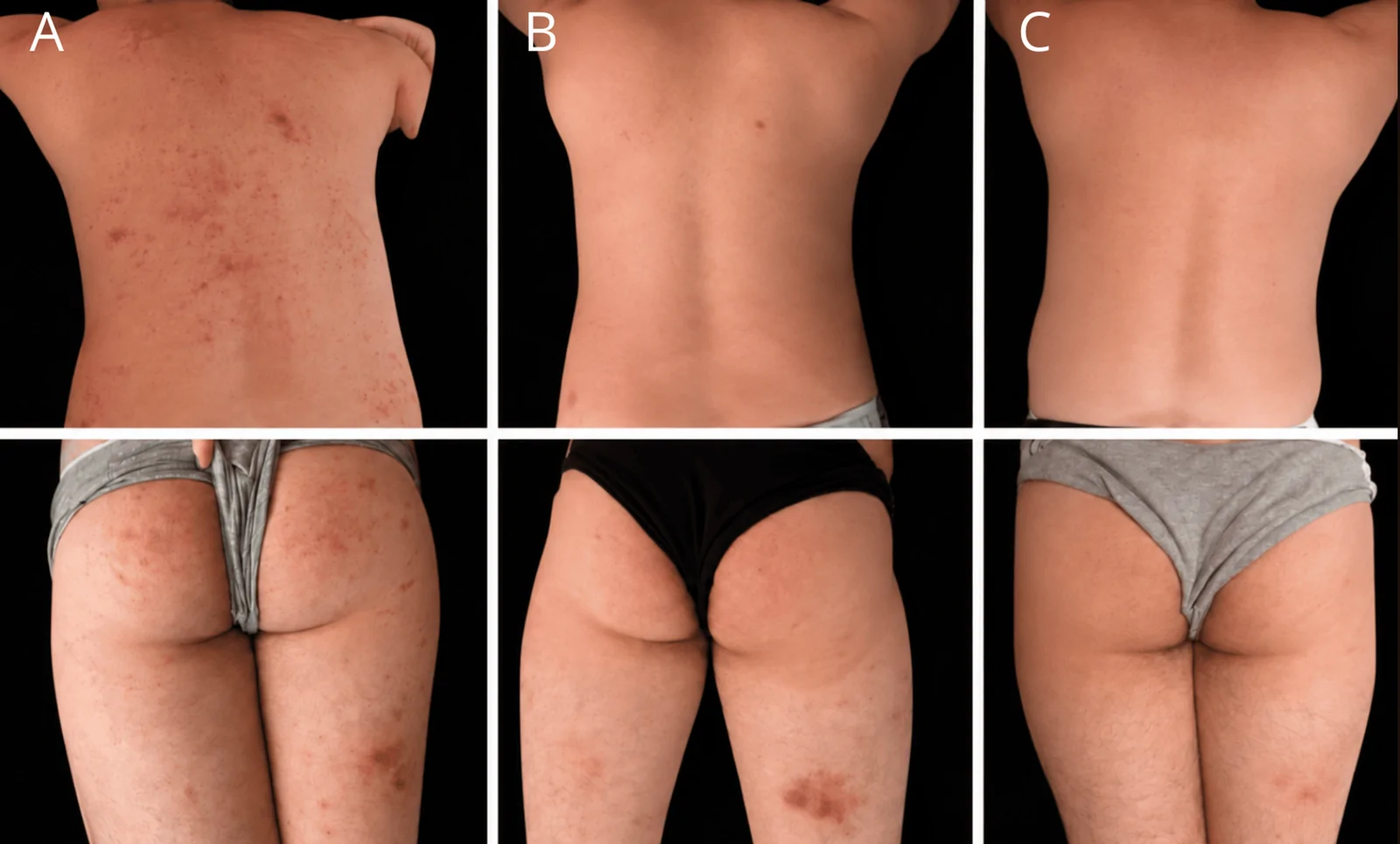
Case 1: Clinical response. (A) Baseline lesions on the back and legs. (B) Week 2: partial improvement. (C) Month 1: clinical improvement during follow-up, as assessed using the EASI. EASI, Eczema Area and Severity Index

Case 2

A 34-year-old woman with a five-year history of AD presented with persistent disease despite multiple previous treatments, including topical and systemic corticosteroids, phosphodiesterase inhibitors, calcineurin inhibitors, and antidepressants. On physical examination, erythematous papules coalescing into a plaque were noted on the dorsal aspects of the hands. At baseline, the EASI score was 20, the NRS score was 8, and the DLQI score was 22. Oral upadacitinib at 15 mg once daily was initiated. At week 2, the EASI score improved by 40%, accompanied by a 50% reduction in the NRS score and a 90.9% reduction in the DLQI score. After one year of follow-up, EASI, DLQI, and NRS scores had decreased by 90%, 100%, and 100%, respectively. No adverse events were reported (Figure 2A, 2B).

**Figure 2:**
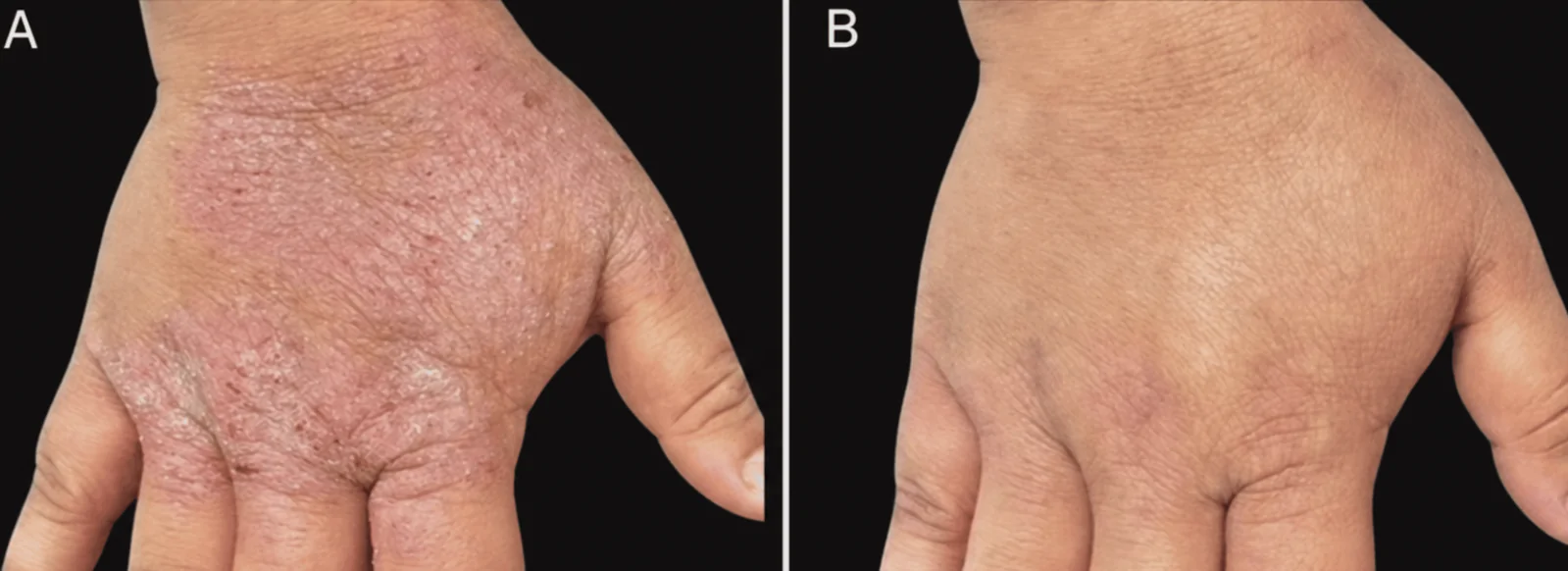
Case 2: Clinical response. (A) Baseline lesions on the dorsal aspects of the hands. (B) Month 1: clinical improvement during follow-up, as assessed using the EASI. EASI, Eczema Area and Severity Index

Case 3

A 42-year-old man with a one-year history of AD had received methotrexate at 15 mg weekly for six months, with limited clinical improvement. Because of persistent disease, a skin biopsy was performed, with histopathologic findings consistent with eczema. On physical examination, erythematous, scaly plaques with lichenification were noted on the palms and soles, with periungual involvement of the hands. Before initiation of upadacitinib, the EASI score was 30, the NRS score was 5, and the DLQI score was 24. Treatment with oral upadacitinib at 15 mg once daily was initiated. By week 2, the EASI score had improved by 43.3%, with an 80% reduction in the NRS score and a 91.7% reduction in the DLQI score. After six months of follow-up, EASI, DLQI, and NRS scores had decreased by 93.3%, 100%, and 100%, respectively. No adverse events were documented (Figure 3A-3D).

**Figure 3:**
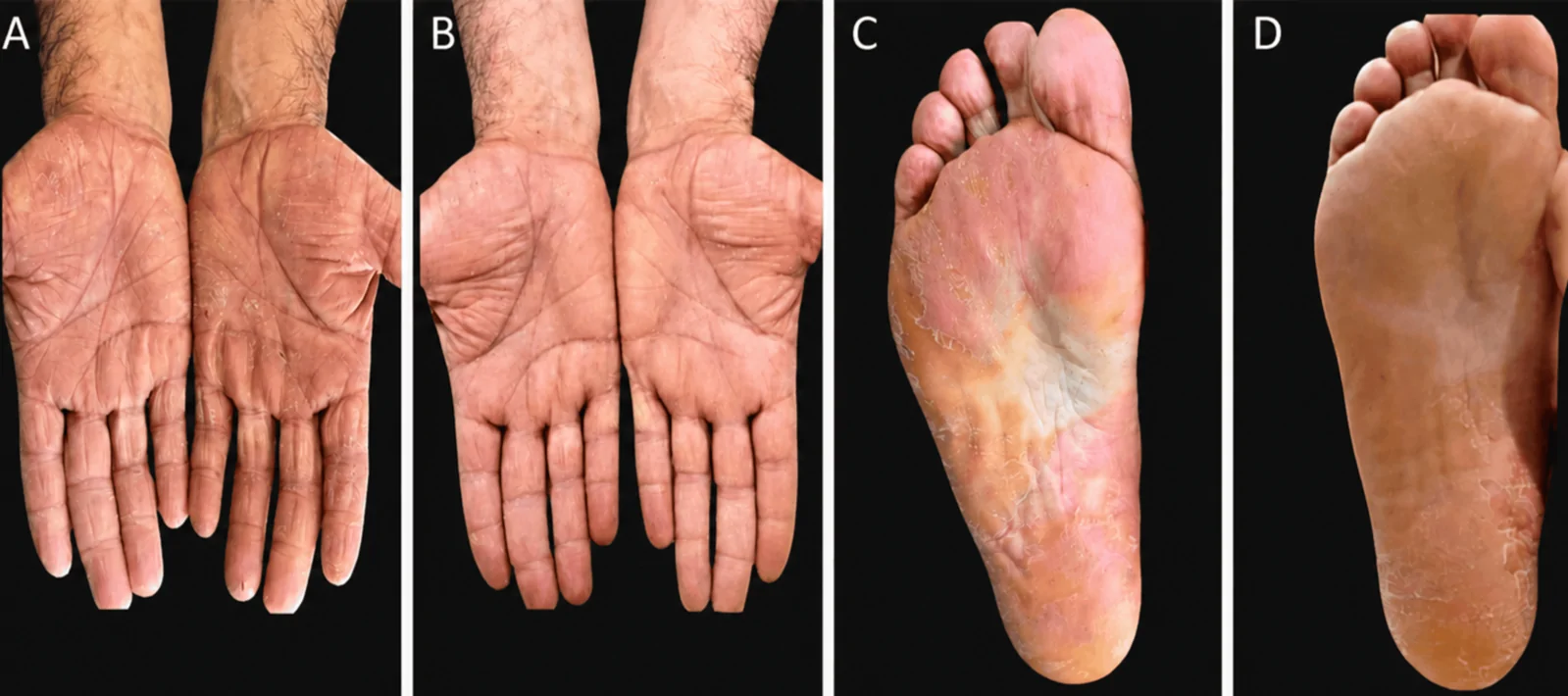
Case 3: Clinical response. (A) Baseline palmar surface. (B) Month 1: palmar surface. (C) Baseline plantar surface. (D) Month 1: plantar surface.

Case 4

A 39-year-old woman had a five-year history of AD and had previously received topical and systemic corticosteroids. She subsequently received dupilumab for approximately 18 months, with good disease control. However, she later experienced a relapse while receiving treatment. On physical examination, a circular, lichenified, erythematous, scaly plaque was noted overlying a nevus of Ota above the right eyebrow, with associated perinasal and nasal erythema. At reassessment, the EASI score was 40, the NRS score was 7, and the DLQI score was 18. The patient also had a concomitant nevus of Ota. Treatment was changed to oral upadacitinib at 15 mg once daily. At week 2, the EASI score improved by 45%, the NRS score decreased by 57.1%, and the DLQI score decreased by 94.4%. After one year of follow-up, EASI, DLQI, and NRS scores had decreased by 92.5%, 100%, and 85.7%, respectively. No adverse events were reported (Figure 4A, 4B).

**Figure 4:**
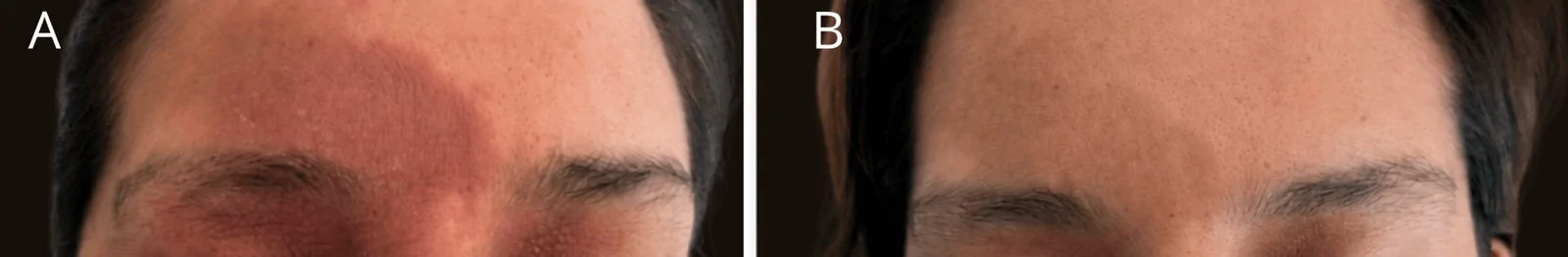
Case 4: Clinical response. (A) Baseline facial lesions. (B) Month 1: clinical improvement during follow-up, as assessed using the EASI. The preexisting nevus of Ota remains visible. EASI, Eczema Area and Severity Index

Case 5

A 50-year-old man presented with a two-month history of AD. Previous treatment included topical corticosteroids and antihistamines. On physical examination, lichenified, erythematous, scaly periungual plaques were noted involving the left thumb, ring finger, and little finger, as well as the right little finger. At baseline, the EASI score was 2.2, the NRS score was 6, and the DLQI score was 7. Treatment with oral upadacitinib at 15 mg once daily was initiated. At week 2, the EASI, NRS, and DLQI scores improved by 54.5%, 66.7%, and 85.7%, respectively. After 1.5 years of follow-up, all three measures had decreased by 100%. No adverse events were reported (Figure 5A, 5B).

**Figure 5:**
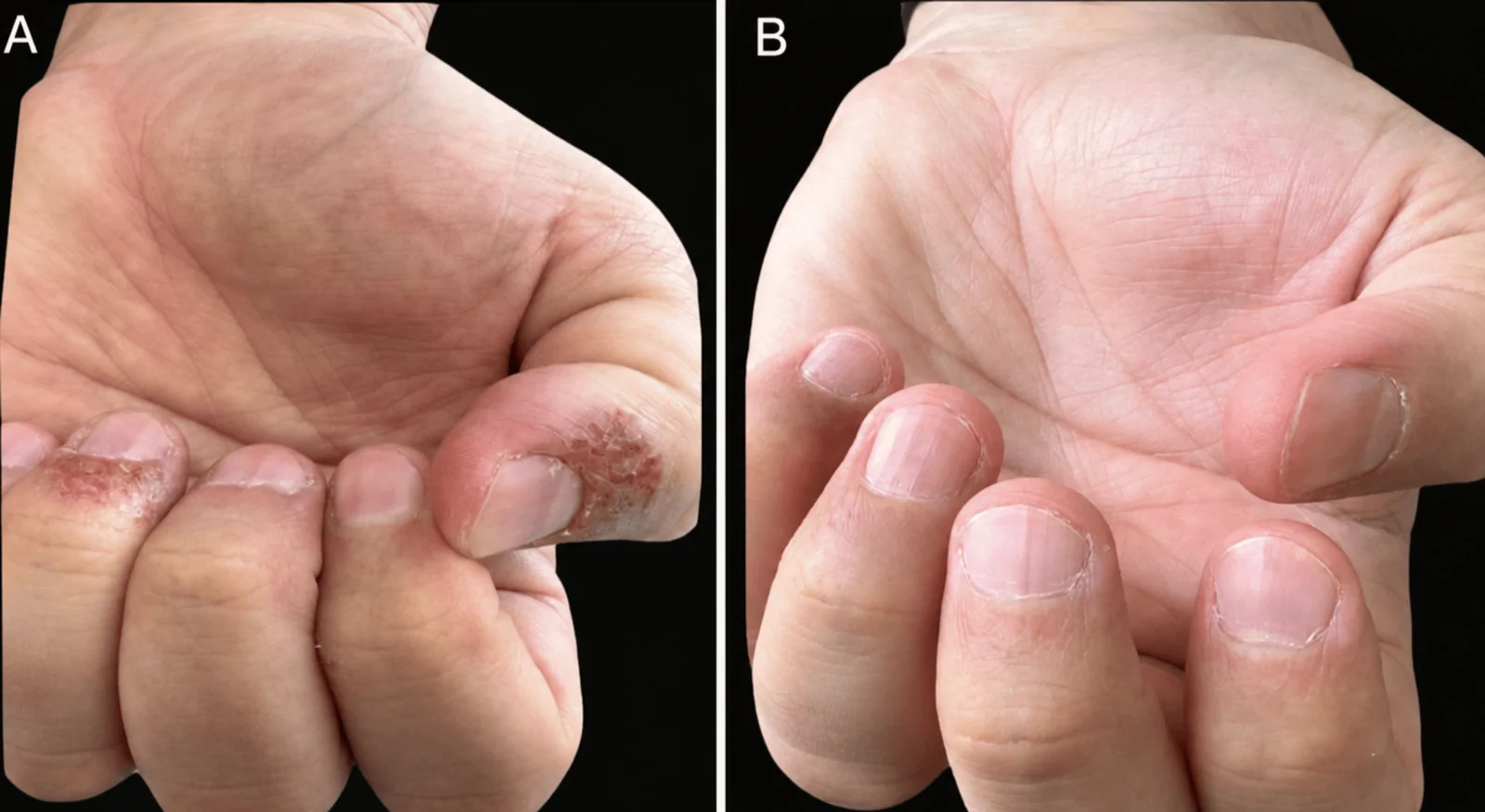
Case 5: Clinical response. (A) Baseline lesions on the fingers. (B) Month 1: improvement during follow-up, as assessed using the EASI. EASI, Eczema Area and Severity Index

Longitudinal changes in disease severity, pruritus, and quality of life were analyzed in the five patients following treatment with upadacitinib (Figures 6, 7).

**Figure 6:**
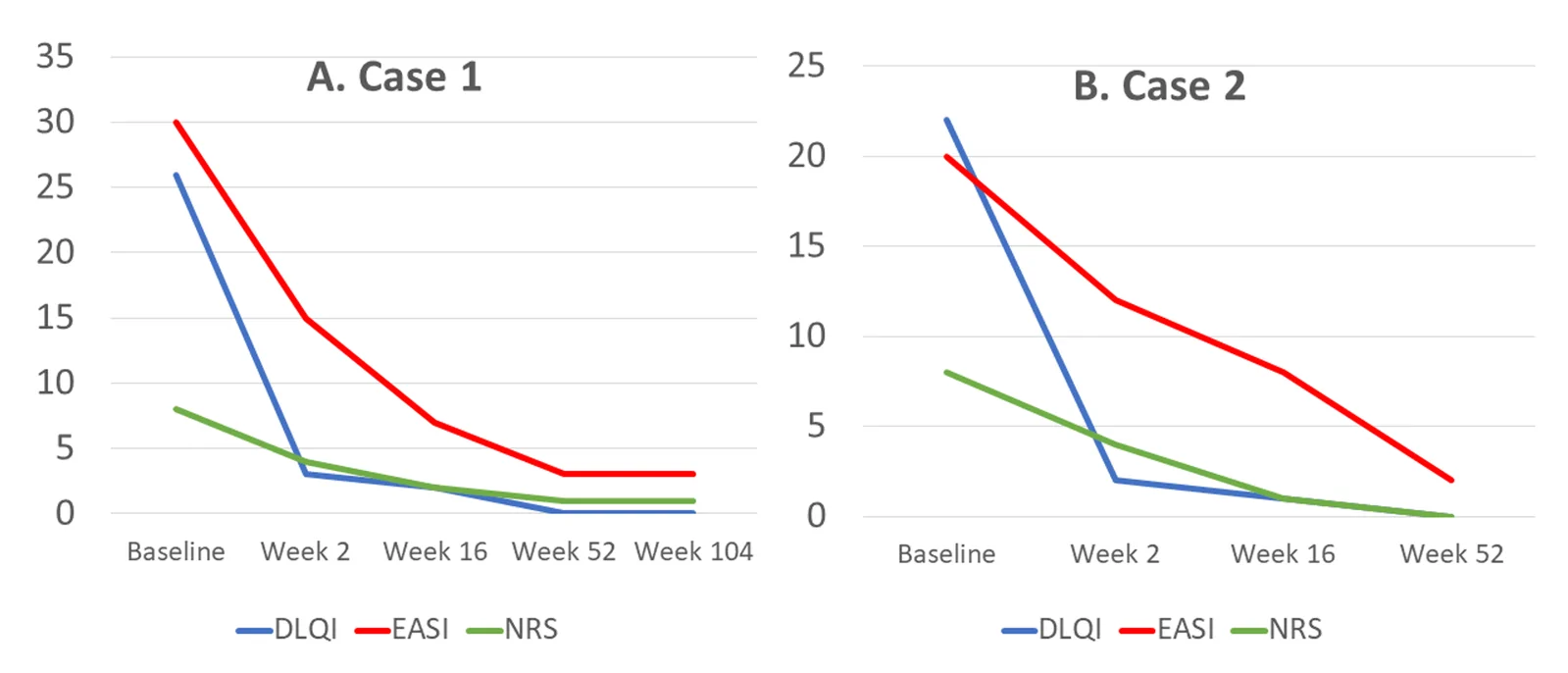
Cases 1 and 2: Longitudinal changes in disease severity, pruritus, and quality of life following treatment with upadacitinib. (A) Case 1: Changes in the EASI, DLQI, and NRS for pruritus from baseline through 104 weeks of follow-up. (B) Case 2: Changes in EASI, DLQI, and NRS from baseline through 52 weeks of follow-up. DLQI, Dermatology Life Quality Index; EASI, Eczema Area and Severity Index; NRS, Numerical Rating Scale

**Figure 7:**
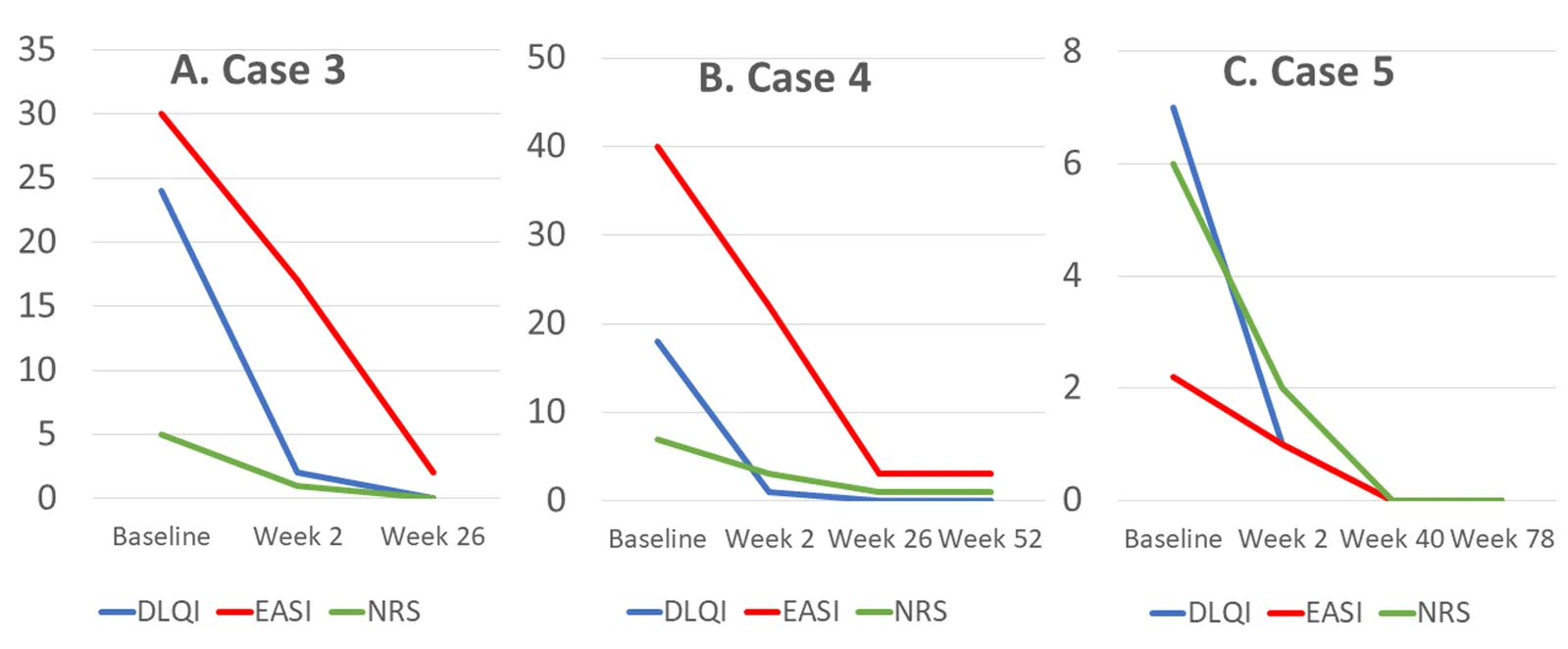
Cases 3-5: Longitudinal changes in disease severity, pruritus, and quality of life following treatment with upadacitinib. (A) Case 3: Changes in the EASI, DLQI, and NRS from baseline through 26 weeks of follow-up. (B) Case 4: Changes in EASI, DLQI, and NRS from baseline through 52 weeks of follow-up. (C) Case 5: Changes in EASI, DLQI, and NRS from baseline through 78 weeks of follow-up. DLQI, Dermatology Life Quality Index; EASI, Eczema Area and Severity Index; NRS, Numerical Rating Scale

## Discussion

AD is a chronic inflammatory disease that can substantially affect the quality of life of patients and their families, representing one of the greatest disease burdens among dermatologic conditions. Its pathogenesis is multifactorial, involving epidermal barrier dysfunction, immune dysregulation, and microbiome alterations, with these processes influenced by complex gene-gene and gene-environment interactions [11].

The pathogenesis of AD involves complex interactions between epidermal barrier dysfunction, type 2 inflammation, and neuroimmune signaling. Several cytokines implicated in these processes, including IL-4, IL-13, IL-22, IL-31, thymic stromal lymphopoietin (TSLP), and interferon gamma (IFN-γ), signal through pathways involving JAK1. Upadacitinib selectively inhibits JAK1, thereby simultaneously reducing signaling from multiple cytokines involved in cutaneous inflammation, epidermal dysfunction, and pruritus. In addition, JAK1-dependent cytokine signaling in sensory neurons contributes to itch, providing a mechanistic basis for the rapid antipruritic effects observed with JAK1 inhibition [6].

Treatment goals have progressively shifted from partial disease control toward sustained skin clearance and meaningful improvement in quality of life. Current treat-to-target recommendations emphasize the simultaneous assessment of clinician-reported disease severity and patient-reported outcomes, with EASI-90 or an EASI score ≤3 and a DLQI score ≤1 representing stringent treatment targets [3].

In this case series of five treatment-experienced patients from Mexico, treatment with upadacitinib was associated with a rapid clinical response. Four patients initiated upadacitinib at 15 mg once daily, whereas one patient initially received 30 mg once daily and was subsequently reduced to 15 mg because of elevated liver enzyme levels. Baseline EASI scores ranged from 2.2 to 40, while DLQI scores ranged from 7 to 26. At week 2, EASI scores decreased to 1-22, corresponding to reductions of 40-54.5% from baseline. NRS scores decreased by 50-80%, while DLQI scores decreased to 1-3 in all patients. These findings indicate early improvement in objective disease severity, pruritus, and quality of life. Clinical responses were maintained throughout follow-up, with all five patients ultimately achieving an EASI score ≤3 and a DLQI score of 0. These findings suggest that upadacitinib may provide a rapid and substantial short-term response in patients with moderate-to-severe AD refractory to topical or biologic therapies [12]. This case series provides real-world clinical observations from a Latin American population, which remains underrepresented in clinical trials (Table 1).

**Table 1:** Clinical characteristics and treatment outcomes of the five patients treated with upadacitinib. DLQI, Dermatology Life Quality Index; EASI, Eczema Area and Severity Index; NRS, Numerical Rating Scale

Characteristic	Case 1	Case 2	Case 3	Case 4	Case 5
Age (years)	33	34	42	39	50
Previous dupilumab treatment	Yes	No	No	Yes	No
Baseline EASI	30	20	30	40	2.2
Week 2 EASI (reduction)	15 (50%)	12 (40%)	17 (43.3%)	22 (45%)	1 (54.5%)
Baseline NRS	8	8	5	7	6
Week 2 NRS (reduction)	4 (50%)	4 (50%)	1 (80%)	3 (57.1%)	2 (66.7%)
Baseline DLQI	26	22	24	18	7
Week 2 DLQI (reduction)	3 (88.5%)	2 (90.9%)	2 (91.7%)	1 (94.4%)	1 (85.7%)
Follow-up (years)	2	1	0.5	1	1.5
EASI at follow-up (reduction)	3 (90%)	2 (90%)	2 (93.3%)	3 (92.5%)	0 (100%)
DLQI at follow-up (reduction)	0 (100%)	0 (100%)	0 (100%)	0 (100%)	0 (100%)
NRS at follow-up (reduction)	1 (87.5%)	0 (100%)	0 (100%)	1 (85.7%)	0 (100%)
Adverse events	Elevated liver enzymes	None	None	None	None

Two patients in our series had previously received dupilumab and initially experienced good clinical responses before developing disease relapse. The difference lies in the mechanism of action and the breadth of pathways inhibited. Dupilumab selectively blocks the shared IL-4/IL-13 receptor, whereas upadacitinib, by inhibiting JAK1, blocks the intracellular signaling of multiple proinflammatory cytokines implicated in AD simultaneously, including IL-4, IL-13, IL-22, IL-31, IFN-γ, and TSLP. This broader inhibition may explain why patients who do not achieve adequate disease control with isolated IL-4/IL-13 blockade may respond well to a drug that simultaneously disrupts multiple pathogenic pathways, including those associated with epidermal hyperproliferation (IL-22), neurogenic pruritus (IL-31), and lesion chronicity (IFN-γ), which are not directly targeted by dupilumab [6,13,14]. This scenario is clinically relevant because patients with AD may require a change in systemic therapeutic strategy when adequate disease control is not maintained. Given that dupilumab and upadacitinib target distinct yet complementary inflammatory pathways, our findings highlight the potential for patients who fail to achieve adequate disease control with dupilumab to experience rapid and clinically meaningful improvement with upadacitinib.

Comparative evidence has demonstrated that upadacitinib provides more rapid and greater improvements in several measures of skin clearance and pruritus than dupilumab [14]. In addition, patients with an inadequate response to dupilumab who subsequently switched to upadacitinib demonstrated clinically meaningful improvements, including clinically meaningful EASI responses [13]. Real-world data further support the effectiveness of upadacitinib in patients previously exposed to dupilumab, including those with an inadequate response to prior biologic therapy [15].

The improvement in DLQI is also clinically relevant. Although EASI provides an objective assessment of disease severity, patient-reported outcomes capture the impact of AD from the patient’s perspective. In our series, all five patients achieved a DLQI score of 1-3 by week 2, despite differences in baseline disease severity. During follow-up, all patients achieved a DLQI score of 0. These findings emphasize the value of incorporating both objective disease severity and patient-reported outcomes when evaluating treatment response [16].

Although no severe adverse events were observed in our five patients, this finding should be interpreted cautiously given the limited sample size. Previous meta-analyses have reported a higher incidence of treatment-emergent adverse events with upadacitinib than with placebo, particularly acne, upper respiratory tract infections, nasopharyngitis, and headache. Most events were mild, manageable, and did not generally lead to treatment discontinuation [17]. In our series, one patient developed elevated liver enzyme levels approximately 1.5 times the upper limit of normal after six months of treatment with upadacitinib at 30 mg once daily. The dose was subsequently reduced to 15 mg once daily, resulting in resolution of the laboratory abnormality, while the clinical response was maintained during follow-up [7].

Limitations

This case series has several limitations. First, the small sample size limits the generalizability of the findings. Second, the descriptive design and absence of a comparator group preclude causal inference and direct comparisons with other systemic therapies. Third, clinical outcomes were assessed during routine clinical practice rather than according to a predefined prospective protocol. Finally, the variability in follow-up duration limits the consistency of long-term outcome assessments. These findings should therefore be interpreted as preliminary real-world observations that warrant confirmation in larger prospective studies.

## Conclusions

This case series highlights the potential role of upadacitinib in the treatment of patients with AD who have persistent disease or experience relapse following previous systemic therapies. All five patients experienced rapid clinical improvement by week 2, with reductions in disease severity and pruritus and improvements in quality of life, followed by sustained disease control during follow-up. All five patients achieved an EASI score ≤3 and a DLQI score of 0 during follow-up. An important observation was the clinical improvement experienced by two patients who relapsed during dupilumab treatment and subsequently switched to upadacitinib. These findings provide real-world clinical observations supporting the potential use of upadacitinib in treatment-experienced patients with AD. However, the small sample size and descriptive nature of this case series preclude definitive conclusions regarding comparative efficacy or safety. Further studies are needed to clarify the role of upadacitinib in this patient population.
